# Environmental Impact of PUR- and Polystyrene-Based Structural Insulated Panels

**DOI:** 10.3390/polym18040518

**Published:** 2026-02-20

**Authors:** Klára Tóthné Szita, Anita Terjék, Viktoria Mannheim

**Affiliations:** 1 Institute of World and Regional Economics, Faculty of Economics, University of Miskolc, 3515 Miskolc-Egyetemváros, Hungary; 2 Institute of Water Resources and Environmental Management, Faculty of Earth and Environmental Sciences and Engineering, University of Miskolc, 3515 Miskolc-Egyetemváros, Hungary; 3 Department of Engineering Management, Faculty of Engineering, University of Debrecen, Ótemető Str. 2-4, 4028 Debrecen, Hungary

**Keywords:** polymer-based insulation, polyurethane foam, life cycle assessment, structural insulated panels, formulation impacts, end-of-life scenarios

## Abstract

Polymer-based insulation materials are widely used to enhance the energy efficiency of buildings; however, their growing application raises concerns related to resource use and end-of-life management. Rigid polyurethane (PUR) foams are key core materials in structural insulated panels due to their favorable thermal and mechanical performance, yet their life cycle environmental impacts—particularly at end-of-life—remain insufficiently quantified. In this study, a cradle-to-grave life cycle assessment (LCA) of PUR-based insulation used in structural insulated panel systems is conducted in accordance with ISO 14040/44 and EN 15804 standards. The assessment is performed using Sphera LCA software (version: GaBi 10.5) and the CML 2016 impact assessment method. Formulation-level variations in rigid PUR foams, including changes in methylene diphenyl diisocyanate content and pentane blowing agent ratio, are explicitly incorporated to evaluate their influence on key environmental impact categories. The results indicate that increasing pentane content leads to higher global warming potential, while this effect may be mitigated or intensified by concurrent changes in diisocyanate content and foam density in fully formulated systems. Three end-of-life scenarios—landfilling, incineration with energy recovery, and mechanical recycling—are analyzed. The findings provide material-level, decision-relevant insights that support environmentally informed formulation strategies and contribute to the development of more circular polymer-based insulation solutions for the built environment.

## 1. Introduction

### 1.1. Global and European Plastic Production

Enhancing the energy performance of buildings is a primary objective in contemporary construction, motivated by climate mitigation goals, escalating energy costs, and stricter regulatory requirements [1]. Thermal insulation materials are critical in reducing operational energy demand; however, their environmental impacts must be assessed across the entire life cycle, including resource consumption, emissions, and waste generation. This consideration is especially important for polymer-based insulation materials, which are primarily derived from fossil fuels and pose significant end-of-life (EoL) management challenges [2,3,4].

**Figure 1:**
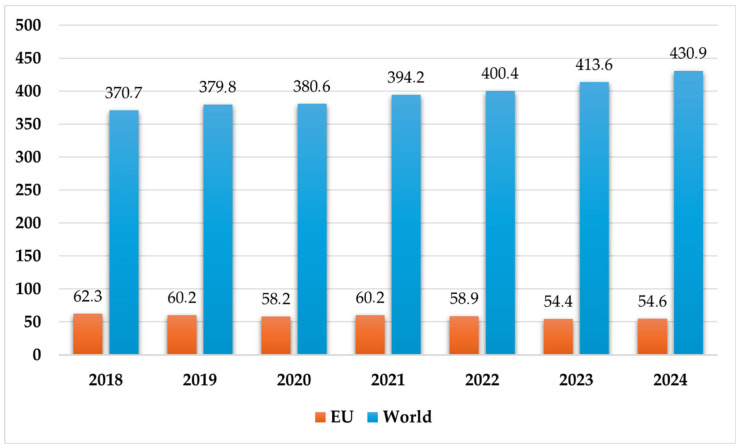
Global plastic production trends over recent decades in megatons (2018–2024). (Own compilation based on the reference [5]).

According to the latest report by Plastics Europe, the global plastic production in 2024 was 430.9 Mt, with 9.5% produced from circular sources. Of this total, the EU 27 region contributed 12.67% (54.6 Mt) [5]. Plastic produced globally through mechanical and chemical recycling reached 41.2 Mt, with the EU 27 region accounting for 19.1% of that amount [5]. Figure 1 illustrates the global increase in plastic production, in contrast to a declining trend in the EU 27+3 region.

Figure 1 shows that global plastic production is increasing, while production in Europe is declining.

In Europe, approximately one-fifth of total plastic demand is associated with construction applications, while recycling rates for construction-related polymer waste remain comparatively low [6].

Polymer components typically represent only a small fraction of construction and demolition waste by mass; nevertheless, their heterogeneity, long service life, and limited recyclability complicate their end-of-life management.

As a result, a significant share of polymer-based construction products is disposed of through landfilling or incineration, often without systematic consideration of alternative recovery pathways [7,8].

**Figure 2:**
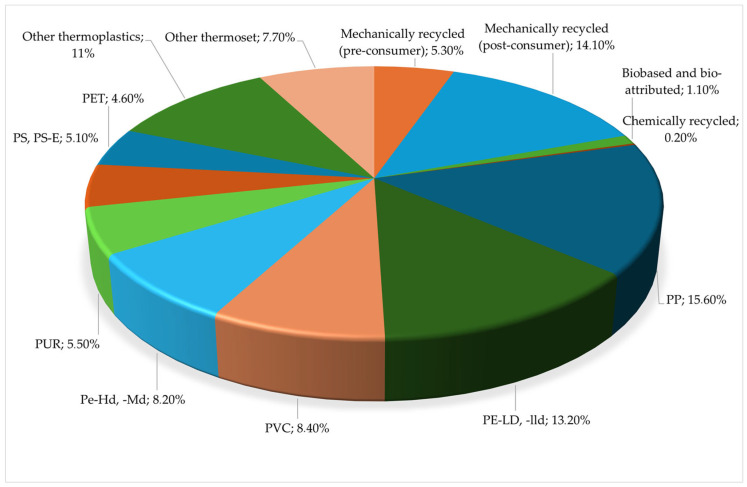
Produced plastic types in the EU 27 region in 2024. (Own compilation based on the reference [5].

Figure 2 shows the produced plastic types in the EU 27 region in 2024.

While Figure 2 indicates that polypropylene is the leading plastic in the European Union with a 15.6 percent share, followed by mechanically recycled (post-consumer) plastic at 14.1% and PE-LD/PE-LLD at 13.2%, it is important to emphasize that rigid polyurethane (PUR) foam is the primary focus of this study. Notably, PUR foam accounted for 5.5% of plastic production in the EU 27 region in 2024 [5].

### 1.2. SIP Technology

In Europe, many family houses have recently been built using lightweight construction technology. In Hungary, this type of prefabricated house is also becoming more popular. Buildings constructed with traditional technology are cost-effective. However, heating costs make up more than 60% of a building’s maintenance expenses. Taking energy-saving solutions and an environmentally conscious approach is now essential [9]. At present, timber-frame solutions dominate. Structural insulated panel (SIP) technology has also emerged. In this system, panels are manufactured and cut to size under controlled conditions. They are made according to production plans prepared by designers. This process reduces waste during construction [10].

The technology, first used overseas in the United States and Canada, later appeared in Japan and Australia. It then spread throughout Europe [11]. Over the years, the process and materials have become more modern and innovative. However, the sandwich structure remains unchanged. As construction trends evolve, this study focuses on SIPs.

This sandwich structure is self-supporting and offers extraordinary stability. Therefore, buildings do not require a separate frame structure. This enables a quick construction process. Panels are made to size, significantly reducing the time needed for structural work [12].

Computer-aided manufacturing now uses automated cutting machines to produce panels from architectural drawings [13]. However, one must consider the environmental impacts over the entire life cycle during production, as required by Regulation (EU) No. 2024/3110. Assessing the environmental impact of construction products is now essential. In the European Union, this must be demonstrated by issuing a Declaration of Performance and Conformity (DoPC) [14]. This process highlights the sustainability of construction products. A more sustainable built environment is key in fighting climate change and moving toward a circular economy [15].

### 1.3. Role of PUR Foams in SIP Systems

Rigid polyurethane foams are among the most widely used polymer-based insulation materials in energy-efficient building solutions [16]. PUR foams are frequently used as the insulating core in structural insulated panel systems, where they are combined with structural facings—commonly oriented strand board (OSB)—to form prefabricated building elements for walls, roofs, and floors. SIP systems integrate structural and thermal functions, enabling high levels of airtightness, reduced thermal bridging, and rapid on-site assembly [17]. While the operational energy benefits of SIP-based construction have been extensively reported, the environmental implications of material composition and PUR insulation end-of-life treatment remain less well addressed [18,19,20].

However, polyurethane and polyisocyanurate (PIR) products are also used as components of structural insulated panels (SIPs). These composite building materials consist of two layers of structural panels separated by an insulating foam layer.

The boards are often made of oriented strand board, and the foam can be expanded polystyrene (EPS), extruded polystyrene (XPS), or polyurethane. Structural insulated panels have excellent insulation properties [21].

SIPs provide better airtightness and superior thermal performance compared to traditional framed walls. Airtightness minimizes infiltration, thereby improving thermal performance. Using SIPs can also significantly reduce heating and air conditioning costs [21].

The air sealing features of SIP houses have led to the creation of the U.S. Environmental Protection Agency’s (EPA) Energy Star program [22,23]. The Energy Star program and SIPs work together to deliver high-performance homes. SIPs have airtightness and superior insulation that meet Energy Star’s strict energy-efficiency standards.

Often, this allows builders to bypass blower door tests and earn the Energy Star label. Builders can qualify by specifying certain insulation components or by referencing the broader Energy Star Homes program. SIPs create insulated, airtight envelopes that reduce energy demand and help homes qualify for Energy Star certification, which is a key goal in green building [22].

### 1.4. Importance of End-of-Life Stage in the Context of Polymer-Based Insulation Materials

Life cycle assessment (LCA) has become an established methodology for quantifying the environmental impacts of construction materials and systems throughout their life cycles, from raw material extraction to end-of-life [24].

Notably, LCA is particularly well-suited to identifying trade-offs across life cycle stages and environmental impact categories, thereby supporting environmentally informed decision-making in material selection and design [19,25].

However, in the context of polymer-based insulation materials, previous LCA studies [26,27] have primarily focused on production-stage impacts or operational energy savings, often treating end-of-life processes using simplified or generic assumptions.

**Figure 3:**
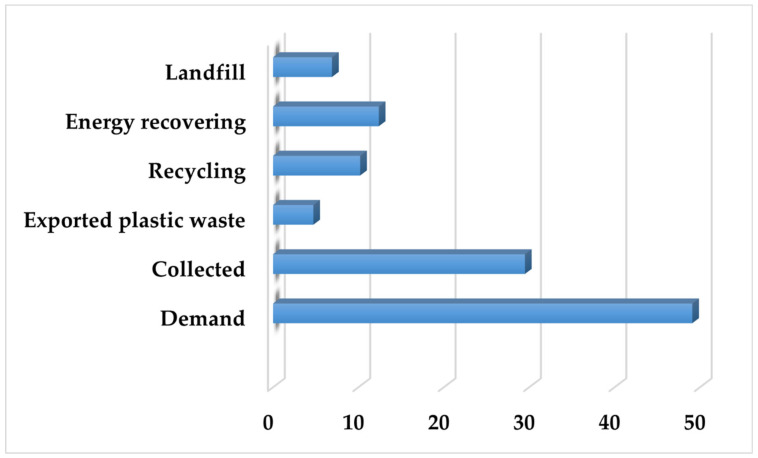
European post-consumer plastic waste in megatons. (Own compilation based on the reference [28]).

Figure 3 shows post-consumer plastic waste in EU 27+3 region in 2020, based on Plastics Europe database [28], an essential factor in planning end-of-life stages.

According to Figure 3, the EU 27+3 average plastic waste treatment in 2020 was as follows: almost 10 Mt recycling of total post-consumer plastic waste (34%), 7 Mt landfilling (24%), and 12 Mt energy recovery (42%) [28].

The end-of-life stage, however, is increasingly recognized as a critical determinant of overall environmental performance, especially in the transition toward a circular economy. For PUR-based insulation materials, realistic end-of-life options include landfill disposal, incineration with energy recovery, and mechanical recycling. Each of these pathways is associated with distinct environmental burdens and potential benefits, which must be evaluated within a consistent methodological framework to ensure comparability.

Furthermore, most existing LCA studies rely on generic database datasets and rarely consider formulation-level variations in PUR foams, such as differences in methylene diphenyl diisocyanate (MDI) content or blowing agent composition, despite their potential influence on environmental impact categories [29].

### 1.5. Research Goal and Scope Definition

The aim of the study is to assess the environmental impacts of rigid polyurethane insulation used in SIP systems. At the same time, the study aims to address these shortcomings by conducting a systematic life cycle assessment of PUR-based insulation used in SIP systems. During the life cycle assessment, PUR formulation-level and SIP-based application assessments are applied using two different functional units.

Following the literature review, in the first step, four PUR samples are analyzed using life cycle assessment to examine eleven impact categories. Here, a special focus is given to determining the global warming potential (GWP) value as a function of changes in pentane content and total content. The primary objective of the environmental life cycle assessment is to investigate the impact of compositional changes in rigid PUR foams on the selected impact categories.

In the second step, a life cycle assessment of three different SIP samples is performed, divided into environmental product declaration (EPD) modules for the cradle-to-grave life cycle of the panels, and, within this, the end-of-life scenario. The analyses concern the determination of the GWP and abiotic depletion potential for fossils (ADPF) values.

Finally, a separate comparative assessment of the end-of-life scenarios is also provided. The analysis evaluates and compares three realistic end-of-life scenarios—landfill, incineration with energy recovery, and mechanical recycling—using a harmonized LCA framework aligned with ISO 14040, ISO 14044, and EN 15804 [30,31,32].

The study’s target audience is researchers, material developers, and designers specializing in polymer-based insulation and prefabricated building technologies.

The research findings aim to support environmentally conscious decisions in material selection and product design for SIP-based building systems. By integrating material composition, building system characteristics, and end-of-life management into a single analytical framework, this study aims to provide decision-making-relevant insights that support the development of more sustainable, circular, polymer-based building solutions.

## 2. Materials and Methods

The objective of this study is to address gaps by conducting a systematic life cycle assessment of PUR-based insulation used in SIP systems. The systematic LCA begins with determining goals and system boundaries. In cases where boundaries are well defined, the next step is to conduct a life cycle inventory analysis (LCI), followed by a life cycle impact assessment (LCIA) [33,34].

During the life cycle assessment, PUR formulation-level and SIP-based application assessments were conducted using two different functional units. The study uses the principles and methods in ISO 14040 and ISO 14044 [30,31]. It also follows EN 15804 [32], including its modular structure and requirements for construction products.

### 2.1. Applied LCA Software

The availability of various databases and software programs enables us to develop solutions for reducing environmental impact at different stages of the life cycle. All life cycle modeling and calculations were performed using Sphera GaBi software (version: 10.5) (Sphera Solutions Ltd., Stuttgart, Germany) [35]. The software enabled consistent modeling of environmental impact assessment across the two assessment levels and for the two functional units, and across the tested end-of-life scenarios. The applied datasets reflect average European production and waste management conditions. The applied LCA software provided valuable resources to support consistent end-of-life modeling. The results from the LCA software highlight the estimated environmental performance across various aspects, including global warming potential and other environmental impacts.

### 2.2. System Boundary

The life cycle system boundaries are defined as cradle-to-grave. The assessment emphasizes the end-of-life stage. The following life cycle stages are included:

- Production stage (A1–A3):

Raw material extraction and processing, production of rigid PUR foam, and manufacturing of SIP components.

- Transport stage (A4):

Transportation of raw materials and finished products using representative European transport datasets.

- Use stage (B1–B7):

The use phase of the SIP systems is included with simplified assumptions. Operational energy performance of the building envelope is not the primary focus. A 30-year service life is assumed for PUR insulation, after which replacement is considered.

- End-of-life stage (C1–C4):

Deconstruction, transport of construction waste, and waste treatment processes corresponding to the examined EoL scenarios.

The benefits and loads beyond the system boundary (Module D) are not explicitly modeled. This maintains comparability across end-of-life scenarios within a consistent allocation framework.

**Figure 4:**
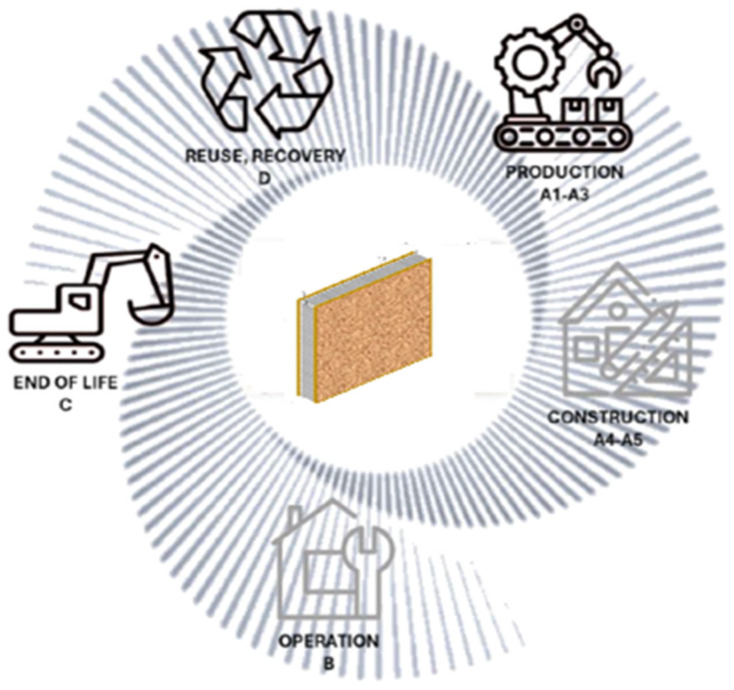
Cradle-to-grave model of SIP. (Authors’ own elaboration, adapted from [36] with permission from the original author).

Figure 4 presents the system boundary of LCA methodology.

### 2.3. Functional Units

In order to implement the PUR-based and SIP-based assessment methods, two different functional units were established to effectively achieve the distinct goals of the study, ensuring targeted and impactful results. All material and energy inputs, emissions, and waste flows are related to the respective functional unit.

1. PUR-based assessment case:

The functional unit is defined as 1 kg of rigid PUR foam produced. This functional unit is applied to evaluate the environmental impacts associated with different PUR formulations, allowing for a direct comparison of formulation-dependent effects independent of the construction application.

2. SIP-based assessment case:

For the evaluation of PUR insulation within SIP systems, the functional unit is defined as 1 m^2^ of SIP element, corresponding to the required thickness of rigid PUR insulation necessary to achieve a target thermal transmittance in accordance with relevant building performance requirements.

### 2.4. Examined End-of-Life Scenarios

Three realistic end-of-life scenarios for rigid PUR insulation were modeled and compared, and are summarized in Table 1.

Chemical recycling routes and bio-based polyol recovery processes were excluded from the present assessment due to limited industrial-scale data availability and insufficiently standardized LCA datasets.

### 2.5. Allocation Rules

Allocation procedures follow the hierarchy defined in ISO 14044 [31]. Where allocation could not be avoided, mass-based allocation was applied, particularly for transportation processes and waste treatment operations. Energy inputs and emissions were allocated proportionally to the material flows associated with the defined functional unit.

Recycling processes were modeled without applying substitution credits to avoid methodological inconsistencies across scenarios and maintain a conservative comparative framework.

### 2.6. Life Cycle Inventory

Life cycle inventory data were compiled using a combination of process-specific input data and background datasets from professional LCA databases. These sources were chosen to ensure a balance between precision and representativeness appropriate for this study. The inventory includes the following:

- Material inputs for rigid PUR foam production, including polyol, methylene diphenyl diisocyanate (MDI), and pentane blowing agents.
- Energy consumption associated with manufacturing processes, based on representative European electricity mixes.
- Transportation processes for raw materials, finished products, and construction waste.
- Emissions to air, water, and soil are generated during production and end-of-life treatment.
- Infrastructure, capital goods, and auxiliary materials with negligible contribution were excluded in accordance with cut-off criteria commonly applied in construction-related LCA studies. This exclusion was made to streamline data collection and focus on elements with substantial environmental impacts.

### 2.7. Life Cycle Impact Assessment Method

The life cycle impact assessment was conducted using the CML 2016 method [37], developed by the Institute of Environmental Sciences at Leiden University. The following impact categories were selected because they are most relevant to the typical environmental concerns associated with polymer-based construction materials, such as resource depletion, emissions, and human health impacts.

Eleven impacts—abiotic depletion (fossil and elements), acidification, eutrophication, global warming, human toxicity, marine and freshwater aquatic ecotoxicities, ozone depletion, terrestrial ecotoxicity, and photochemical ozone creation—were used in this assessment. The global warming potential is set at 100 years, except for biogenic carbon.

The formation of tropospheric ozone was considered in terms of its potential to generate photochemical ozone. Human toxicity potential describes the effects of harmful substances. Abiotic resource depletion is one of the most debated impact categories. Table 2 presents the examined impacts.

The normalization and weighting methods were consistent across all analyses. The normalization reference used represents the environmental impacts of European Union countries. The LCIA survey in 2012 used the CML 2016 method for Europe.

## 3. Results and Discussion

### 3.1. Environmental Performance of PUR Formulations

The LCA results for the manufacturing phase of PUR formulations indicate that formulation-level variations lead to measurable, though moderate, differences across the examined environmental impact categories.

Figure-based results (see corresponding figures) show that none of the investigated formulations exhibits extreme deviations; however, consistent trends can be identified with respect to raw material composition.

Table 3 presents the formulations of PUR-based prototypes, with recipe names indicating their compositions.

Among the assessed impact categories, marine aquatic ecotoxicity potential (MAETP) represents the dominant contribution for all PUR formulations, accounting for approximately one-third of the total normalized environmental burden.

This result is primarily attributable to upstream emissions associated with polyol and isocyanate production, which are known to involve resource-intensive chemical processes and emissions affecting aquatic ecosystems. In addition, abiotic depletion of fossil resources constitutes the second most significant contributor, reflecting the fossil-based origin of the main PUR constituents.

**Figure 5:**
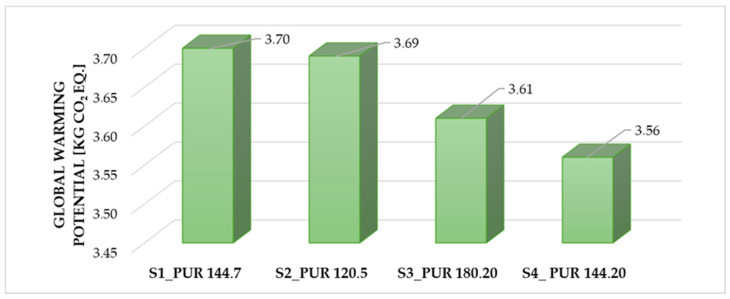
Global warming potential values of PUR samples [kg CO_2_ eq.]. (Authors’ own elaboration).

Figure 5 shows a clear decreasing trend in the measured values from the S1_PUR sample to the S4_PUR sample.

Based on the results of Figure 5, S1_PUR and S2_PUR exhibit the highest and nearly identical GWP values (3.70 and 3.69 kg CO_2_ eq.), indicating a higher environmental impact. In contrast, S3_PUR, and especially S4_PUR, show lower GWP values, reflecting a reduced contribution to global warming.

Overall, the results demonstrate a decreasing GWP trend from S1_PUR to S4_PUR, suggesting an improved environmental performance in the latter samples.

**Figure 6:**
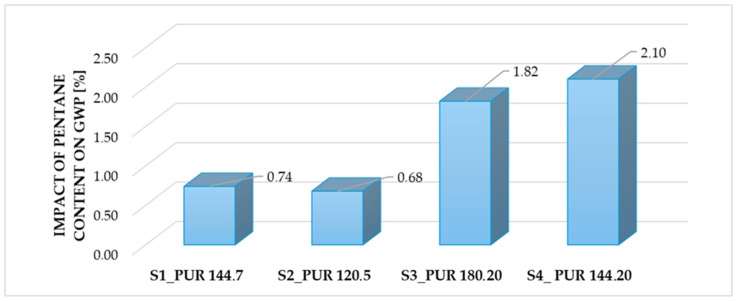
Percentage impact of pentane content on the PUR samples’ global warming potential. (Interpretation: from left to right: S1, S2, S3 and S4). (Authors’ own elaboration).

Figure 6 illustrates the impact of pentane content on the global warming potential of the PUR samples in percent.

According to Figure 6, from S1 to S4, a clear increasing trend in GWP can be observed. S1 and S2 samples show the relatively low impact of pentane content on the GWP (0.74 and 0.68%), while S3 and S4 exhibit a substantial rise, reaching 1.82 and 2.10%, respectively. This indicates that, when considered independently, higher pentane content increases the global warming potential of the PUR samples.

However, in full formulation systems, this effect may be offset or amplified by concurrent changes in MDI content and overall foam density, as reflected in the combined results shown in Figure 5.

It should be noted that Figure 4 and Figure 5 present different comparative perspectives. Figure 5 shows how simultaneous changes in the methylene diphenyl diisocyanate (MDI) content and the pentane blowing agent ratio influence the properties of the PUR formulations.

In contrast, Figure 6 focuses solely on how changes in pentane content affect GWP. Consequently, the apparent discrepancy between the two figures does not indicate a contradiction, but rather highlights the interaction effects between formulation parameters.

**Figure 7:**
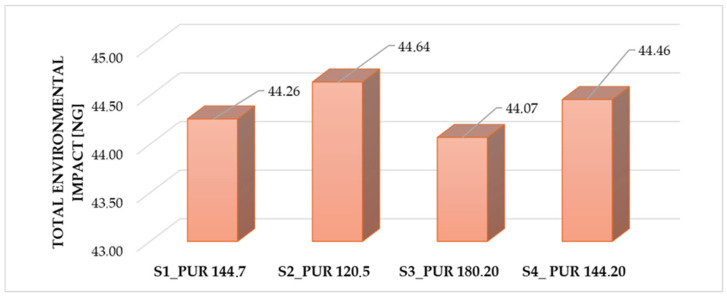
Normalized and weighted environmental impact of samples (ng/kg sample). (Authors’ own elaboration).

Figure 7 compares the magnitude of total environmental impact per PUR formulation expressed in nanograms.

The results of Figure 7 show relatively small differences among the formulations, with values ranging from approximately 44.07 to 44.64 ng. PUR 120,5 exhibits the highest environmental impact, while PUR 180,20 shows the lowest value, indicating a slightly reduced environmental burden. Overall, the data indicate that although the formulations differ in composition, their environmental impacts are broadly comparable, with only minor variations between samples.

**Figure 8:**
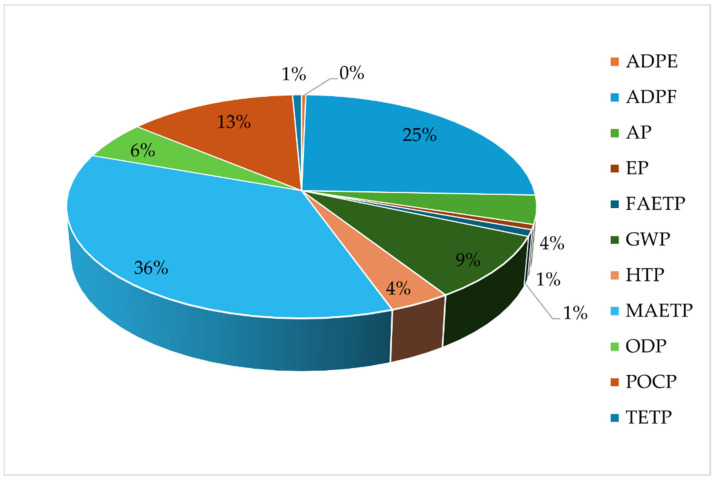
Average normalized and weighted impacts of PUR samples. (Authors’ own elaboration).

If the normalized and weighted average value for each examined effect is calculated for the four PUR samples, then the dominance of the effects among the effect categories can be observed. Figure 8 presents the averaged impacts.

Based on the average of the normalized and weighted values, it can be seen that toxicity potential dominates the environmental impacts, with damage to the marine ecosystem accounting for 36%, human toxicity (HTP) for 4%, and terrestrial and freshwater toxicity potentials (TETP and FAETP) for the remaining 1%. Fossil abiotic depletion accounts for 25%, photochemical ozone (POCP) for 13%, GWP for 9%, ozone depletion (ODP) for 6%, acidification (AP) for 4%, and eutrophication (EP) for 1%.

Variations in methylene diphenyl diisocyanate (MDI) content influence several impact categories, particularly global warming potential and fossil resource depletion. Formulations with higher MDI content tend to exhibit higher GWP values, which can be explained by the energy-intensive synthesis routes used to produce aromatic isocyanates.

In contrast, increasing the proportion of pentane as a blowing agent at a constant polyol–MDI ratio results in a slight reduction in GWP. This trend is associated with pentane’s lower embedded energy compared to alternative blowing agents, as well as its influence on foam density and material efficiency.

These results show that environmental performance at the formulation level cannot be linked to a single component. Instead, the global warming potential of rigid PUR foams depends on how the isocyanate content, blowing agent ratio, and material efficiency interact. Therefore, optimizing PUR formulations for the environment requires integrated assessments rather than single-parameter trends.

Overall, the results indicate that formulation optimization alone cannot fundamentally alter the environmental profile of rigid PUR insulation, but it can contribute to incremental improvements. These findings support the view that formulation-level decisions should be considered complementary to, rather than a substitute for, optimized end-of-life management strategies. Therefore, isolated trends observed for individual formulation parameters should not be interpreted independently from their interaction with overall foam composition and density.

### 3.2. Environmental Performance of Structural Insulated Panels

SIP systems are known for their strong environmental performance. These systems consist of an insulating foam core sandwiched between two structural facings, typically made of OSB or metal. The primary environmental benefits of SIP systems include the following [38,39,40]:

- Energy Efficiency: SIPs provide superior insulation compared to traditional framing methods, reducing heating and cooling energy consumption.
- Reduced Waste: the manufacturing process of SIPs generates less waste than that of conventional building materials, making them a more sustainable option.
- Lower Carbon Footprint: by improving energy efficiency, SIP systems can help reduce greenhouse gas emissions throughout a building’s lifetime.
- Sustainable Materials: many SIPs are made from renewable resources or recycled materials, contributing to more sustainable building practices.

Overall, SIP systems can significantly improve the environmental performance of buildings compared to traditional construction methods [41,42].

Traditional SIPs, typically comprising an insulating foam core enclosed between oriented strand board facings, are reviewed alongside nontraditional FRP-faced (fiber-reinforced polymer-faced) SIPs. These panels can be made from a variety of insulation materials, the most common of which are expanded polystyrene, polyurethane foam, and mineral wools (rock wool, glass wool). EPS is also available in a graphite version.

ElastoPIR insulation material has excellent fire protection properties due to its high-temperature stability, low thermal conductivity (l), and flame resistance. It is made with fewer flame retardants and less bromine than traditional versions. The latter’s preliminary design considerations, emphasizing optimized foam core thickness and density to enhance thermal insulation without compromising structural integrity under diverse load conditions, are explored [21,43,44,45,46].

**Figure 9:**
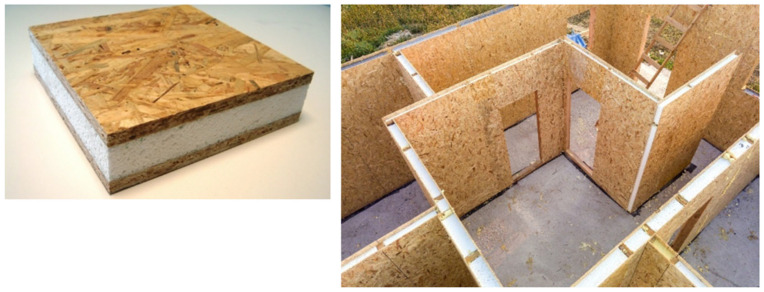
Schematic structure of a structural insulated panel consisting of OSB facings and a rigid PUR insulation core (source: this photo was purchased from Shutterstock—shutterstock.com—as part of our previous IS-Sus Con/Spread of Innovative Solution for Sustainable Construction project).

Figure 9 presents the schematic structure of a SIP consisting of OSB facings and a rigid PUR insulation core.

When rigid PUR insulation is evaluated in the context of structural insulated panel systems, its environmental impacts are distributed across multiple life cycle stages. If these life cycle stages are examined using the environmental product declaration technology modules, then the following can be concluded:

- The production stage (A1–A3) remains a major contributor to overall impacts, driven by the manufacturing of PUR foam and OSB facings. Transportation (A4) contributes marginally under the applied European average assumptions and does not significantly influence comparative outcomes.
- The use stage (B1–B7) shows limited direct environmental relevance within the system boundaries of this study, as operational energy savings are not explicitly credited. This conservative modeling choice ensures that differences observed in the results are primarily attributable to material composition and end-of-life treatment, rather than to assumptions about building operation.
- At the end-of-life stage (C1–C4), separating and processing PUR insulation from SIPs is technically challenging, underscoring the importance of evaluating realistic waste-management pathways. The integration of PUR insulation into SIP systems does not fundamentally alter the relative importance of impact categories observed at material level; however, it emphasizes the relevance of end-of-life treatment given the composite nature of the building element.

The available EPD for the SIP element’s environmental impact differs because the SIP uses various components. These include different board panels, insulation elements (EPS, PUR, mineral wool), additive materials, thin layers, and mass [47].

Table 4 and Table 5 show the GWP and ADPF of environmental product declarations (EPDs). Table 4 and Table 5 contain the GWP and ADPF values of SIP elements made with three different insulating materials (SP-170 panel/SIP 3000 mm × 1250 mm × 170 mm with EPS insulation core, SIPA B6.5/SIP—blank 6.5” with XPS foam core, and S-P-07434/smart panel with PUR insulation core), projected per 1 m^2^, with the same insulating layer thickness.

Based on Table 4 and Table 5, it is clear that in the A1–A3 phase, the SIP element containing PUR has the lowest GWP value, while the SIP containing XPS shows the highest value. According to the aggregate GWP, the EPS element has a GWP more than three times that of the PUR panel (62.2 kg CO_2_ eq. versus 17.56 kg CO_2_ eq.) due to its high GWP content in the C4 phase.

In terms of fossil resources, the SIP containing EPS requires the most (431 MJ), while PUR-based SIPs require two orders of magnitude less.

### 3.3. Theoretical Recycling Solutions for Construction PUR/PIR

In principle, reuse is possible if the dismantled PUR/PIR boards were mechanically fixed, the dismantling was selective and the boards are clean and undamaged. However, this is rare. Mechanical recycling is common because the technology is simple, the foams can be used in various composites after grinding [48,49,50].

During chemical recycling, the products decompose under controlled conditions, and the polyol can be recovered and reused to produce foams. Chemical recycling encompasses various processes such as glycolysis, hydrolysis, and amino lysis, as well as thermochemical and biological degradation. This type of recycling aims to recover the original raw materials, particularly high-quality recycled polyol monomers, which are necessary for producing a new polymer with the same characteristics [51,52,53,54]. Chemical recovery is also possible during pyrolysis, but it is an energy-intensive process [55,56].

Energy recovery and landfilling are dominant solutions, particularly if chemical/mechanical recycling operates with a high logistical burden and a low-quality recovered fraction [57].

Rigid polyurethane and polyisocyanurate foams are highly effective thermal insulation materials for building envelopes, but their end-of-life management (especially as demolition waste) is a major obstacle to their circular application.

PUR/PIR foams typically have a thermoset structure, so “classical” melt recycling is not a viable option; currently, recovery typically ends up in energy recovery or landfill, while preserving the material value (e.g., polyol recovery) is only possible with targeted technologies and appropriate waste quality [58].

In the case of PIR, depolymerization is generally even more difficult due to the higher isocyanate index and isocyanurate rings, which pose a separate EoL challenge [59,60].

Factors affecting the environmental performance of recycling depend on the following:

- Selective dismantling, separate collection and contamination;
- Facings, adhesives, and coatings to reduce the purity of the fraction;
- Additives, flame retardants, and regulatory compliance to reduce usability.

### 3.4. Comparative Evaluation of End-of-Life Scenarios

The comparative evaluation of the examined EoL scenarios demonstrates that waste management strategy selection has a pronounced influence on the environmental performance of PUR-based insulation materials [20]. Landfill disposal, incineration with energy recovery, and mechanical recycling exhibit clearly distinct impact profiles across the analyzed environmental categories.

It should be noted that EPD module-based results primarily reflect direct emissions associated with specific life cycle stages and do not capture long-term resource loss or circularity-related aspects, which are essential for interpreting landfill performance from a sustainability perspective.

Landfill disposal shows relatively low normalized impact values in several categories within the applied LCA framework; this outcome, however, primarily reflects methodological characteristics of commonly used landfill datasets, which typically account for limited direct emissions while insufficiently representing long-term environmental burdens and permanent material value loss. From a circular economy perspective, landfill disposal remains the least favorable option, as it results in irreversible resource loss and offers no recovery potential [61]. In addition, long-term emissions beyond the modeled time horizon are not fully represented in conventional landfill datasets, which may lead to an underestimation of toxicity-related impact.

Incineration with energy recovery exhibits a mixed environmental profile. Combustion-related emissions contribute significantly to climate change and toxicity-related impact categories, while partial compensation may occur through recovered energy. The overall environmental performance of incineration is highly sensitive to modeling assumptions. These include energy substitution, background energy mix, and allocation rules. In credit-free or conservative modeling approaches, incineration may therefore exhibit higher normalized impacts than landfill in several categories, highlighting the importance of transparent methodological choices in EoL assessments.

Module D was intentionally excluded from the assessment to ensure methodological consistency and to avoid scenario-dependent substitution assumptions, thereby enabling a conservative and transparent comparison of end-of-life options.

Mechanical recycling consistently shows the most favorable environmental performance among the examined scenarios across most impact categories. By enabling the reuse of PUR waste as a secondary raw material, mechanical recycling reduces the demand for virgin material production and avoids upstream environmental burdens [62]. Mechanical recycling does not restore the polymer to its original quality, but the results show that even downcycling pathways provide meaningful environmental benefits compared to disposal options.

Although this pathway represents a downcycling process and does not restore the original polymer quality, the results demonstrate that even limited material recovery can provide meaningful environmental benefits compared to disposal-oriented options.

Taken together, the findings indicate that differences between end-of-life scenarios exert a stronger influence on environmental performance than formulation-level variations in PUR foams, particularly for impact categories related to resource depletion and toxicity. This underlines the critical role of end-of-life strategy selection in improving the sustainability of PUR-based insulation systems.

### 3.5. Implications for Circular Economy and Design Practice

The results of this study highlight the critical role of end-of-life considerations in the environmental assessment of polymer-based construction materials. While rigid PUR insulation offers clear benefits during the use phase of buildings, its overall sustainability performance is strongly conditioned by the chosen waste management pathway.

From a circular economy perspective, mechanical recycling emerges as the most favorable option among the currently realistic end-of-life scenarios for PUR insulation in SIP systems. However, the feasibility of recycling is closely linked to product design, dismantlability, and waste separation practices. This finding emphasizes the importance of design-for-disassembly and material compatibility in prefabricated construction systems [7,8].

Formulation-level optimization can further support circular design strategies by marginally reducing environmental burdens during production, but it cannot compensate for environmentally unfavorable end-of-life routes. Consequently, integrating end-of-life considerations into early design—alongside formulation choices—represents a key leverage point for improving the environmental performance of PUR-based SIP systems.

## 4. Conclusions

This study presents a comprehensive life cycle assessment of PUR-based insulation used in structural insulated panel systems, with a particular focus on formulation-dependent effects and end-of-life management strategies. By applying a harmonized LCA framework aligned with ISO 14040, ISO 14044 and EN 15804 standards [30,31,32], the environmental performance of PUR insulation was systematically evaluated across multiple impact categories and life cycle stages.

The results clearly demonstrate that end-of-life management plays a decisive role in shaping the overall environmental profile of PUR-based insulation. Among the evaluated scenarios, landfill disposal consistently exhibited the highest environmental burdens, particularly in toxicity- and resource-related impact categories. Incineration with energy recovery showed intermediate performance, offering partial compensation through energy recovery but remaining associated with combustion-related emissions.

In contrast, mechanical recycling emerged as the environmentally preferable option across most impact categories, confirming that even downcycling-oriented recovery pathways can deliver meaningful environmental benefits compared to disposal-oriented approaches.

In addition to end-of-life strategies, formulation-level variations in rigid PUR foams were found to influence selected environmental impacts, most notably global warming potential and fossil resource depletion. Increased methylene diphenyl diisocyanate (MDI) content tended to raise the global warming potential, while higher pentane ratios contributed to moderate reductions. Although these differences were less pronounced than those associated with end-of-life treatment, the results indicate that formulation optimization can support incremental environmental improvements when combined with appropriate waste management strategies.

From a design and decision-making perspective, the findings emphasize that material formulation choices alone are insufficient to substantially improve environmental performance if unfavorable end-of-life pathways are applied. Instead, integrating end-of-life considerations at the early design stage, together with formulation-sensitive LCA, represents a key leverage point for enhancing the sustainability of SIP-based construction systems. In this regard, mechanical recycling potential, dismantlability, and material compatibility should be considered alongside thermal and mechanical performance criteria.

The study is subject to certain limitations. The analysis relies on Sphera software database-based inventory data and average European energy and waste management conditions, which may influence the absolute magnitude of the calculated impacts. Furthermore, advanced chemical recycling technologies and bio-based polyol pathways were excluded due to limited data availability and insufficient industrial-scale implementation. The two functional units used in this study serve distinct analytical purposes and are not intended for direct numerical comparison; rather, they support complementary interpretation at the material and system levels.

Future research should extend the proposed framework to include emerging recycling technologies, regionally differentiated scenarios, and dynamic service-life assumptions in order to further support circular design strategies for polymer-based construction materials.

Overall, this work contributes to the growing body of LCA-based research on polymer insulation materials by providing a system-level, end-of-life-oriented assessment of PUR-based SIP systems. The results offer decision-relevant insights for material developers, designers, and policymakers aiming to reduce environmental burdens and promote more circular solutions in the construction sector.

## Figures and Tables

**Table 1:** End-of-life scenarios.

	Short Description	Longer Description
Scenario 1	Landfilling	PUR insulation waste is transported and disposed of in a controlled landfill as mixed construction plastic waste.
Scenario 2	Incineration with energy recovery	PUR insulation waste is treated in a waste incineration facility with energy recovery, following average European waste-to-energy datasets.
Scenario 3	Mechanical recycling	PUR insulation waste undergoes mechanical size reduction and processing, enabling its reuse as filler material in secondary polyurethane products.

**Table 2:** Examined environmental impacts based on the Sphera software database [35].

Impact Category	Equivalent
Abiotic depletion potential for fossils, ADPF	MJ
Abiotic depletion potential for elements, ADPE	kg Sb eq.
Acidification potential, AP	kg SO_2_ eq.
Eutrophication potential, EP	phosphate kg eq.
Freshwater aquatic ecotoxicity potential, FAETP	kg DCB eq.
Global warming potential, GWP 100 years	kg CO_2_ eq.
Human toxicity potential, HTP inf.	kg DCB eq.
Marine aquatic ecotoxicity potential, MAETP	
Ozone depletion potential, ODP	R11 eq.
Photochemical ozone creation potential, POCP	kg ethene eq.
Terrestrial ecotoxicity potential, TETP	kg DCB eq.

**Table 3:** PUR-based prototype samples with the different polyol–MDI–pentane contents.

Sample Markings and Meanings	Polyol (*w*/*w*%)	MDI (*w*/*w*%)	Pentane (*w*/*w*%)
S1_PUR = PUR 144,7 (reference material)	100	144	7
S2_PUR = PUR 120,5	100	120	5
S3_PUR = PUR 180,20	100	180	20
S4_PUR = PUR 144,20	100	144	20

**Table 4:** Summary table of GWP of EPDs (unit: kg CO_2_ eq.).

Sample Name	A1–A3	C1	C2	C3	C4	D
SP-170 SIP	20.8	0.01970	0.223	0	41.20	−0.0095
SIPA B6.5	23.3	0.00045	0.173	0	1.27	0
S-P-07434	13.8	0.02000	0.227	0	3.50	0.0100

**Table 5:** Summary table of ADPF of EPDs (unit: MJ).

Sample Name	A1–A3	C1	C2	C3	C4	D
SP-170 SIP	466.00	341.00	1.76	1.26	0	−379.00
SIPA B6.5	272.00	0.0035	0.36	0	0.598	0
S-P-07434	1.07	0.2620	2.94	0	0.400	−9.21

## Data Availability

The original contributions presented in this study are included in the article. Further inquiries can be directed to the corresponding author.
